# New Strategies in Diagnosis and Treatments for Brain Tumors

**DOI:** 10.3390/cancers15112879

**Published:** 2023-05-23

**Authors:** Sven Kantelhardt

**Affiliations:** Department of Neurosurgery, Vivantes Hospital im Friedrichshain, Landsberger Allee 49, 10249 Berlin, Germany; sven.kantelhardt@vivantes.de

In general, cancer is one of the most frequent causes of death. According to WHO estimations, cancer is responsible for 9.6 million deaths in a single year worldwide (2018 [1]). The 2019 Global Burden of Disease (GBD) study estimated that about 1 million patients worldwide suffer from brain tumors [2]. Among these malignant gliomas, which account for about 81% of malignant brain tumors [3], is especially terrifying. Reasons for this are the frequent and rapid neurological deterioration, and the relatively young age of patients affected [4]. Diagnosis and treatment have a tremendous impact on the quality of life of patients and families. Although cancer research is generally one of the main and best funded topics in health science, life expectancy of glioma patients has been rather stagnating in recent years [5].

While no definite breakthrough in glioma therapy has been achieved yet, recent progress in various directions of glioma research fuel hopes for better treatment regimes. Of special interest are the fast-evolving field of bioinformatics and the application of artificial intelligence. The combination of several innovative approaches might finally converge towards a more individual, personalized therapy, reducing side effects and improving prognosis.

Self-renewal and differentiation of glioma stem cells have been identified as underlying mechanisms for the formation, progression, and recurrence of malignant glioma. Molecular mechanisms and pathways, determining the biological behavior of these glioma stem cells and the development of glioma subtypes, are accordingly of high importance [3]. This research does not only enhance our understanding of glioma in general but helps to identify possible candidates as prognostic markers and targets for molecular or immune therapy.

Thus Park et al., using immunofluorescence in glioma cell lines and FDG-PET in patients, showed that NADPH oxidase 2-induced high glycolytic activity provokes the expression of mesenchymal-subtype-related gene COL5A1. Promoting the development of the mesenchymal subtype of GBM [6].

Sun et al. demonstrated that nicotinamide N-methyltransferase (NNMT) overexpression levels correlate with tumor cell progression in glioma cell lines and clinical samples from 59 patients, which, again, provides a possible prognostic biomarker [7].

Supporting the efforts in classical laboratory work, bioinformatics, and by processing big data can help to analyze and recognize complex interrelationships. Thus Zolotovskaia et al. applied bioinformatics to reconstruct the FREM2 pathway and assess its activity (the FREM2 gene codes for an integral membrane protein involved in cellular calcium regulation). The authors showed that FREM2 molecular pathway activity was a better biomarker for progression-free and overall survival than FREM2 gene expression levels; for example, that the application of bioinformatics can add valuable information to conventional lab results [8].

The application of bioinformatics algorithms, likewise, allows the analysis of big datasets. This leads to a somewhat reverse approach compared to conventional laboratory work on specific molecules or pathways. Belotti et al. investigated the role of neurotransmitters (NTs) in GBM. They identified genes coding for NT receptors in the dataset. Interestingly, these genes were progressively downregulated with the increasing aggressiveness of the glioma to which the individual dataset corresponded. They further found a strong negative correlation with genes associated with immune response and established cancer hallmark genes. This suggests that NTs have a cancer-inhibiting ability and possible therapeutic value [9].

In a comparable approach, Sorokin et al. applied whole transcriptome sequence profiling to identify novel prognostic markers in GBM. They found overexpression of the non-coding RNA CRNDE in all GBM datasets. When comparing GBM patients with different clinical courses, overexpression was significantly associated with poor overall- and progression-free survival [10]. This bioinformatics approach, which can simultaneously analyze the entire transcriptome, will help to achieve an enhanced understanding of complex interactions, which are difficult to understand when concentrating on specific molecules or pathways. It further carries the potential of suggesting effective therapies on an individual basis [11].

In terms of diagnostics, several studies using intraoperative (5-ALA induced) fluorescence and PET examinations [12,13] show that MRI-contrast enhancement is not always and/or does not completely match tumor boundaries. While conventional histopathology remains the gold standard, researchers have been looking for other means to delineate tumor margins more precisely and more reliably in a non-invasive fashion. MRI techniques, for example, using FLAIR sequences [14], are among the most promising options. Kinoshita et al. compared MRI relaxometry to identify tumor tissue by assessing of cell density to conventional ^11^C-Mehtionine PET. The results were validated by stereotactic biopsy and histopathologic workup. They showed that T1-relaxation times correlated well with MET-PET [15].

The definition of tumor boundaries is, however, not only of diagnostic importance. High-precision radiotherapy and surgical resection guidance likewise rely on the exact delineation of tumor margins. Bütof et al., therefore, investigated whether biological tumor characteristics can be used to identify tumor margins in individual samples from an orthotopic brain tumor model. The aim was to optimize the clinical target volume (CTV) for irradiation therapy. They used mass spectrometry (MALDI) to identify biological tumor characteristics and correlated the finding to the detection of established stem cell and invasion markers in histopathology. As the results matched well, they concluded that a CTV definition based on biological tumor characteristics is possible [16]. It is clear that malignant glioma compromises not only a heterogeneous group of tumors but that individual tumors show a wide variety of molecular properties in themselves [17]. The authors suggest that visualization of heterogeneities within the tumor could be potentially used to define radiotherapy-sensitive and resistant areas [16].

It has been demonstrated that the effect of irradiation on glioma cells can be enhanced by combination with radio-sensitizing chemotherapy [18]. A synergistic effect that can also be used the other way around. In a recent report, Li et al. describe the enhanced effect of the tyrosine kinase inhibitor Anlotinib, if administered under radiotherapy, which might open up the blood–brain barrier [19]. However, Brüning-Richardson et al. assessed the radio-sensitizing effect of pharmacologic GSK-3 inhibition on established and patient-derived GBM cell lines. They found a significant effect and suggested that the applied substance (AZD2858) carries a high potential to maximize the efficiency of irradiation [20].

However, the delineation of tumor margins is not the only radiological challenge in GBM diagnostics. The early and reliable identification of recurrent glioma is likewise of great therapeutic relevance. Qin et al. reviewed and discussed the value of different imaging and post-processing techniques. While various MRI sequences provide different perspectives, focusing on cell density [15], cell proliferation, blood perfusion, brain metabolism, etc., to discriminate tumor progression and other changes. Paralleling the current situation in basic research, the application of radiomics and artificial intelligence is increasingly applied in (radiological) diagnostics with considerable success [21].

Apart from imaging studies, such as MRI and PET examinations, blood tests offer further opportunities to enhance non-resp. low invasive diagnostics in gliomas [22]. Cabezas-Camarero et al. performed a study on plasma-based liquid biopsy in 10 glioma patients. They used next-generation sequencing to preoperatively identify IDH1 mutations in primary glioma. While the false negative rate was rather high, at 86%, the clinical sensitivity was 100%. This opened an interesting perspective in this emerging field of glioma diagnostics [23].

Despite all the progress made, therapeutic interventions do have a downside. Recent studies have shown that they are drivers for molecular changes and differentiations within these tumors that help tumor cells to evade these therapies [17,24,25]. Therefore, treatment regimes effective on the initial tumor might be rendered insufficient in the recurrent situation. Immune therapy seems a promising target, especially in gliomas pre-treated with irradiation and/or alkylating substances, such as Temozolomide [17]. Paret et al. observed that medulloblastoma (the most common glioma in children) presents few immunogenic targets. This, they believe, is a consequence of the rather low mutation rate in these tumors. Their data suggests, however, that immunogenic fusions, such as the EPC2-GULP1 fusion, might provide alternative targets for immunotherapeutic approaches [26].

Eventually, malignant gliomas recur even following successful initial treatment. Frequently applied therapies in recurrent glioma include Temozolomide re-challenge, nitrosourea-based therapy (Lomustine), or agents targeting neo-angiogenesis (Bevacizumab) [27]. Unfortunately, none of these therapies could claim general superiority [28,29,30]. Thus, no commonly accepted standard exists for this situation [31]. Therefore, the search for hitherto unnoticed factors that might contribute to an effective second-line therapy is still open. CMV infection is one of these factors. It has previously been shown to be involved in the progression of GBM [32]. Accordingly, the antiviral drug Valganciclovir was applied in primary GBM. Due to the small size of the study, it failed to meet the primary endpoint, while showing a better prognosis in an exploratory analysis [33]. Pantalone et al. now presented a retrospective series on 29 patients with recurrent GBM in which Valganciclovir was administered as an add-on to a second- or third-line therapy. These patients likewise showed prolonged survival in comparison to the control [27].

The Cancers Special Issue titled “New Strategies in Diagnosis and Treatments for Brain Tumors” is comprised of eleven original articles and one review. The aim of the volume is to provide an overview of recent developments in general understanding, prognosis, diagnostics, and therapy of malignant gliomas. The articles were published between July 2021 and December 2022.
